# Transcriptome Analysis of Stigmas of *Vicia faba* L. Flowers

**DOI:** 10.3390/plants13111443

**Published:** 2024-05-23

**Authors:** Inés Casimiro-Soriguer, David Aguilar-Benitez, Natalia Gutierrez, Ana M. Torres

**Affiliations:** Área de Mejora Vegetal y Biotecnología, IFAPA Centro Alameda del Obispo, Apdo. 3092, 14080 Cordoba, Spain; david.aguilar@juntadeandalucia.es (D.A.-B.); natalia.gutierrez.leiva@juntadeandalucia.es (N.G.); anam.torres.romero@juntadeandalucia.es (A.M.T.)

**Keywords:** faba bean, transcriptome, stigma, style, stigmatic cuticle, stigma receptivity

## Abstract

Pollination in angiosperms depends on complex communication between pollen grains and stigmas, classified as wet or dry, depending on the presence or absence of secretions at the stigma surface, respectively. In species with wet stigma, the cuticle is disrupted and the presence of exudates is indicative of their receptivity. Most stigma studies are focused on a few species and families, many of them with self-incompatibility systems. However, there is scarce knowledge about the stigma composition in Fabaceae, the third angiosperm family, whose stigmas have been classified as semidry. Here we report the first transcriptome profiling and DEGs of *Vicia faba* L. styles and stigmas from autofertile (flowers able to self-fertilize in the absence of manipulation, whose exudate is released spontaneously) and autosterile (flowers that need to be manipulated to break the cuticle and release the exudates to be receptive) inbred lines. From the 76,269 contigs obtained from the de novo assembly, only 45.1% of the sequences were annotated with at least one GO term. A total of 115,920, 75,489, and 70,801 annotations were assigned to Biological Process (BP), Cellular Component (CC), and Molecular Function (MF) categories, respectively, and 5918 differentially expressed genes (DEGs) were identified between the autofertile and the autosterile lines. Among the most enriched metabolic pathways in the DEGs subset were those related with amino acid biosynthesis, terpenoid metabolism, or signal transduction. Some DEGs have been related with previous QTLs identified for autofertility traits, and their putative functions are discussed. The results derived from this work provide an important transcriptomic reference for style-stigma processes to aid our understanding of the molecular mechanisms involved in faba bean fertilization.

## 1. Introduction

In angiosperms, pollination depends on complex communication between the male (pollen grains) and the female (stigma/style) reproductive organs. In the compatible pollen–pistil interaction, several events are involved: pollen capture, adhesion, germination, penetration of pollen tube into the stigma, growth of the pollen tube through the style, and final entry of the pollen tube into the ovule. Stigmas can generally be classified into two main groups according to the presence (wet stigmas) or absence (dry stigmas) of a viscous secretion on the stigma surface [1,2]. Once pollen grains are transferred to the stigma by abiotic (e.g., water, wind) or biotic vectors (e.g., insects, birds), or directly by contact between the anther and the stigma, pollen–pistil interactions differ between species. In species with wet stigma (e.g., *Nicotiana tabacum*, *Lilium longiflorum*), the cuticle is disrupted due to the presence of exudates, which can be composed by lipids, proteins, carbohydrates, phenols, glycoproteins, ions, and enzymes, such as esterases and peroxidases [3,4]. Unspecific pollen grains adhere to the stigma surface thanks to exudates, and pollen hydration occurs passively, transferring water from the stigmatic exudates [5]. By contrast, in species with dry stigmas (e.g., *Arabidopsis thaliana*, *Zea mays*, *Oryza sativa*), the events following pollination are species-specific and highly regulated [6]. Pollen adhesion and germination have been well studied in species with self-incompatibility systems such as Brassicaceae, Poaceae, or Papaveraceae, and a high diversity of molecules and processes have been discovered (reviewed in [4,7]).

Previous proteomic and transcriptomic studies in species with wet and dry stigmas indicate that both strategies express unique as well as common genes and proteins during stigma maturation. It is expected that the evolution of genes involved in sexual reproduction occurs at a higher rate than those in charge of background processes; moreover, those genes responsible for the maintenance of species boundaries will be species-specific and, therefore, different between species [5,8]. Allen et al. [9] found that certain gene families were consistently found in pistil tissues of different species such as cytochrome P450, ATP-binding cassette (ABC) transporters, lipid transfer proteins (LTPs), zinc finger proteins, extensin-like proteins, receptor protein kinases, disease resistance proteins, or nodulin/mtn3 genes. Similarly, Sang et al. [10] found, at a broad level, that the proportion and abundance of stigma proteins in different functional categories (e.g., ‘defense and stress response’, ‘carbohydrate and energy metabolism’, ‘protein metabolism and folding’) were similar between maize (dry) and tobacco stigmas (wet), indicating that, in general, similar processes occur in both types of stigmas. However, the specific proteins found in ‘signal transduction’ and ‘lipid metabolism’ categories showed low protein homologies between wet and dry stigmas [10].

Fabaceae is the third largest plant family after Asteraceae and Orchidaceae [11] that is found to be globally distributed, but for which few stigma composition studies have been carried out [12,13,14]. Most studies performed so far have been focused on a few species and families such as maize, rice, *Lilium*, *Arabidopsis*, *Brassica*, *Crocus*, *Petunia*, and *Nicotiana*. Allen et al. [9], conscious of this reality, added a new clade to the pool of studied species: *Senecio squalidus*. It belongs to the Asteraceae family and possesses a ‘semidry’ stigma, which shows intermediate characteristics between dry and wet stigmas. This condition is characterized by having secretory cells with exudate retained by cuticle or protein pellicle that can be ruptured by pressure or physical friction [15,16]. The stigma of the Fabaceae has been classified as wet or semidry, although some cases of dry stigmas have been reported (e.g., *Cassia grandis*, *Caesalpinia echinata*) [17]. The semidry stigma is particularly characteristic of the Papilionoideae subfamily [17], which comprises ~14,000 species [18]. Some of its members are economically and culturally important legume crops such as pea, lentil, chickpea, and faba bean. Legumes fix atmospheric nitrogen into available ammonia, promoting the nitrogen fertilization of natural soils. Many of them are used for food or forage because of their high content in protein, starch, fiber, and other essential nutrients, but they can also be exploited for industrial processes (dyes, gums) or have medicinal properties [19].

In a global climate change context, it is expected that the reproductive success of plants, including those involved in agriculture, will be affected [20,21]. In addition to physiological alterations caused by abnormal climatic conditions, the reproductive success of entomophilous plants can be also affected by changes in plant–pollinator interactions such as variations in the population distribution of pollinators or the uncoupling of flowering phenology and insect life cycles [22,23,24]. Hence, it is important to extend the knowledge about the mechanisms that promote self-fertilization, since pollinator dependence could restrict plant reproduction under climate change scenarios.

Faba bean (*Vicia faba* L.) is a partially allogamous species, with both cross- and self-fertilization happening in the same plant [25]. Cross-fertilization depends on pollinator activity, and an unstable yield as well as low fruit and seed sets are related to low visitation rates. On the other hand, self-fertilization occurring by spontaneous selfing could ensure pod and seed set in the absence of pollinators [26,27,28]. The ability of a flower to self-fertilize in the absence of pollinators or mechanical disturbance is termed autofertility [29]. The degree of autofertility differs among faba bean genotypes, and it has been related to some floral features like wider style–ovary angle, shorter style, shorter stigmatic papillae, few and shorter stylar hairs, thinner intervening cuticles with rupture previous to anthesis, or lower quantities of pollen grains [27,30,31]. Despite the importance of the rupture of the stigmatic cuticle for successful fertilization in faba bean flowers, little is known about the underlying processes taking place on the stigmas. Recently, a highly saturated genetic map was built, and several quantitative trait loci (QTLs) associated with different autofertility traits were detected. Some of the QTLs were related to the rupture of the stigmatic cuticle in chromosomes I and VI, although the function of the associated marker was not clearly related with autofertility [32].

Advances in faba bean breeding have been slow and costly due to their large genome (13 Gbp) and mixed breeding system. RNA-Seq analysis is a relatively inexpensive method and provides data for single-nucleotide variations, clarifying transcriptional and post-transcriptional gene regulation and transcript rearrangements. Differentially expressed genes (DEGs) can be identified with this method to facilitate our in-depth understanding of key biological and physiological mechanisms. Although some comparative transcriptomic analyses have been performed in faba bean using different tissues to understand stress responses such as drought [33], frost [34], or disease resistance [35], no transcriptional information on the genes involved in the fertilization process is available, and the molecular basis of this essential process is still unknown. Herein, we have performed the first transcriptome analysis of the styles and stigmas of faba bean flowers from lines contrasted for autofertility and combined this information with previous QTL analyses for autofertility traits with three main objectives: (i) amplify the genetic information available for stigmas in a different plant species and family, (ii) identify differentially expressed genes (DEGs) between autofertile and autosterile lines to better understand the functional biology underlying this important trait, and (iii) overlay these DEGs on the previous QTLs to identify the candidate genes associated with autofertility.

## 2. Results

### 2.1. Transcriptome Sequencing and De Novo Assembly

A total of 1,189,079,630 raw reads were obtained from the 18 libraries. After quality control and filtering, the total number of reads was 1,077,199,910. A summary of the transcriptome de novo assembly data is shown in Table 1. The assembly of sample AF27.18 in Trinity produced 76,269 contigs with an N50 of 2387 bp and an average contig length of 982.9 bp.

### 2.2. Annotation and Differential Expression Analysis

From the 76,269 contigs of the whole transcriptome, 45.1% of the sequences (34,421 contigs) could be annotated with at least one GO term against the PLAZA 4.5 dicots database in TRAPID, and 34,379 of them were assigned to 7720 gene families. In addition, 29.1% of the contigs showed full-length or quasi full-length sequences, although more than 55% of the transcripts provided no information.

**Figure 1:**
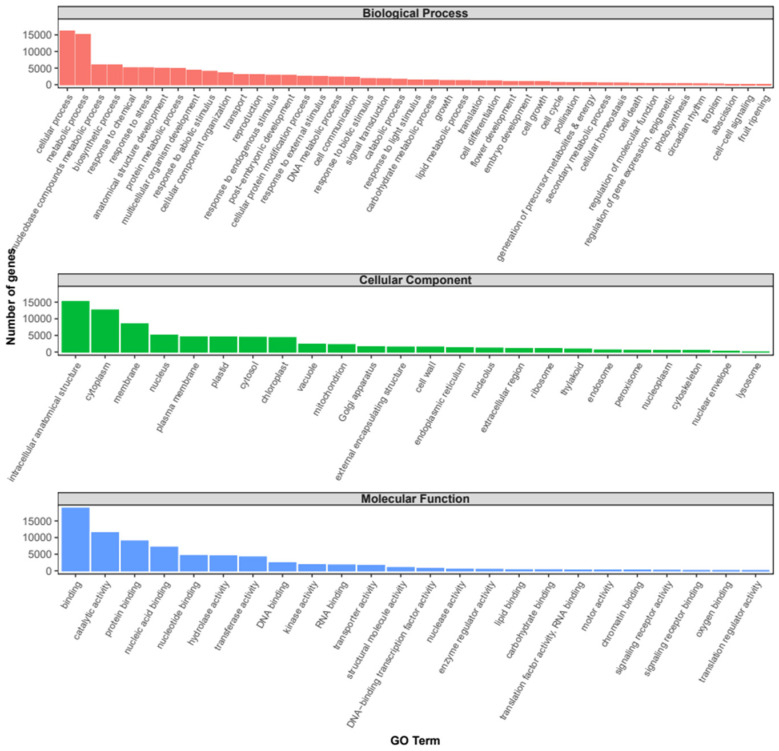
Gene ontology (GO) functional classification of the *V. faba* transcriptome obtained from stigma and style samples. Histogram of the main transcripts annotated to specific GO categories: Biological Processes, Cellular Components, and Molecular Function. The *x*-axis represents the GO term, and the *y*-axis represents the number of genes annotated.

Gene ontology analyses retrieved a total of 8439 different GO terms assigned, which were summarized according to the GO slims categories for the plants in Figure 1. A total of 115,920, 75,489, and 70,801 annotations were assigned to Biological Process (BP), Cellular Component (CC), and Molecular Function (MF) categories, respectively. The top two within BP were ‘cellular process’ and ‘metabolic process’. Some other terms revealed by the analysis were ‘nucleobase-containing compound metabolic process’, ‘response to stress’, ‘anatomical structure development’, ‘reproduction’, response to different stimuli, or ‘transport’. Among the CC category, ‘intracellular’ followed by ‘cytoplasm’ and ‘membrane’ were the most abundant terms. Regarding the MF category, the majority of contigs were annotated within ‘binding’ and ‘catalytic activity’. In the binding category, ‘protein binding’, ‘nucleic acid binding’, ‘nucleotide binding’, ‘DNA binding’, and ‘RNA binding’ were the most abundant categories. On the other hand, ‘hydrolase activity’, ‘transferase activity’, ‘kinase activity’, and ‘transporter activity’ were also important categories (Figure 1).

**Figure 2:**
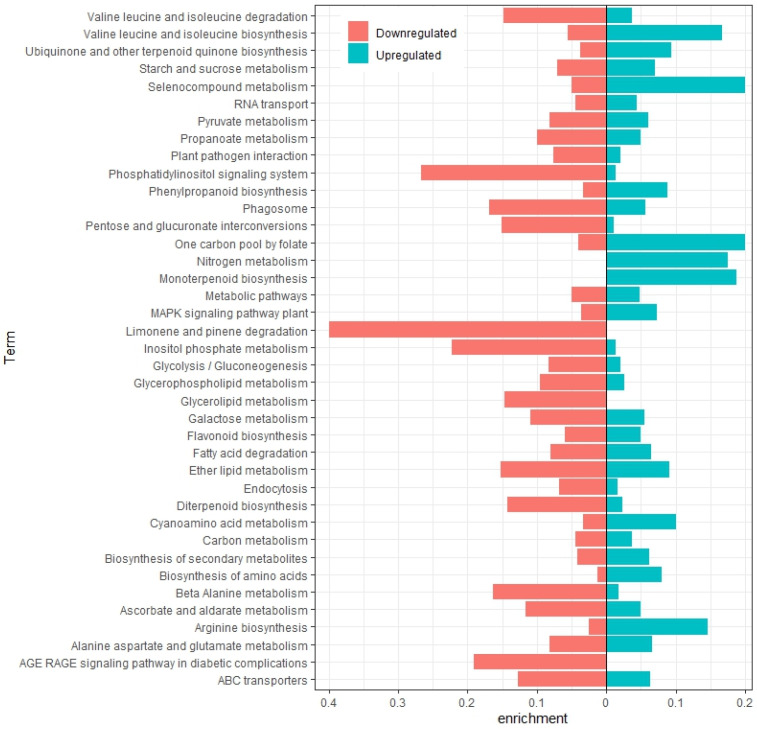
Metabolism pathway assignments of the downregulated (red, left) and upregulated (blue, right) differentially expressed genes (DEGs) in AF vs. AS based on the Kyoto Encyclopedia of Genes and Genomes (KEGGs). The enrichment degree is calculated compared to the number of genes for a certain category present in *Medicago truncatula* in the KOBAS-i database.

The differential expression analyses performed in edgeR revealed 5918 differentially expressed genes (DEGs) between the autofertile lines (AF) and the autosterile lines (AS). Of them, 3443 genes were upregulated (higher expression values in AF than in AS) and 2475 genes were downregulated (with significant lower expression values in AF than in AS) (Supplementary File S1). The KEGGs pathway enrichment analysis using KOBAS-i indicated that the up- and downregulated genes were significantly enriched in 39 functional groups, with ‘Biosynthesis of secondary metabolites’, ‘Metabolic pathways’, and ‘Starch and sucrose metabolism’ being the most significantly enriched terms in both groups. Upregulated genes were enriched in ‘Selenocompound metabolism’, ‘One carbon pool by folate’, ‘Monoterpenoid biosynthesis’, ‘Nitrogen metabolism’, or biosynthesis of certain amino acids like arginine, valine, leucine, and isoleucine. On the other hand, downregulated genes were particularly enriched in ‘Limonene and pinene degradation’, ‘Phosphatidylinositol signaling system’, ‘Inositolphosphate metabolism’, ‘AGE-RAGE signaling pathway in diabetic complications’, ‘Phagosome’, ‘ABC transporters’, or ‘Glycerolipid metabolism’ (Figure 2). Some of these significant KEGG terms were exclusive of up- or downregulated genes. Thus, ‘Nitrogen metabolism’ and ‘Monoterpenoid biosynthesis’ were exclusive of upregulated genes, whereas ‘Limonene and pinene degradation’, ‘AGE-RAGE signaling pathway in diabetic complications’, and ‘Glycerolipid metabolism’ were exclusive of the downregulated genes (Figure 2).

The GO annotation analysis of the DEGs between AF vs. AS performed in TRAPID showed that 2802 out of 5918 contigs (47.3%) were annotated, with at least one GO term and 2793 transcripts being assigned to 1285 gene families. Based on the GO slims categories for plants, the general GO term classification of the DEGs showed a similar distribution to the one exhibited by the whole transcriptome (Supplementary File S2). Thus, ‘cellular process’ and ‘metabolic process’ were the most abundant subcategories in BP, ‘intracellular’, ‘cytoplasm’, and ‘membrane’ were the most abundant in the CC, and ‘binding’ and ‘catalytic activity’ were the most abundant in the MF categories. 

The GO enrichment analysis performed in TRAPID showed that more than 60, 10, and 50 GO terms were significantly enriched in the BP, CC, and MF categories, respectively (Supplementary File S3). Among the BP component, the most enriched general terms for the upregulated genes in AF vs. AS were ‘glycoside metabolic process’, ‘aminoglycan metabolic process’, ‘chitin metabolic process’, ‘glucosamine-containing compound catabolic process’, ‘cell wall macromolecule catabolic process’, ‘nucleotide catabolic process’, ‘salicylic acid catabolic process’, or ‘lignin catabolic process’ with log2 enrichment values > 2. In the downregulated genes, GO terms like ‘negative Rho protein signal transduction’, ‘negative regulation of Ras protein signal transduction’, ‘pollen tube adhesion’, ‘protein homotetramerization’, or ‘phosphatidilinositol-mediated signaling’ were the ones showing log2 enrichment values > 2. In the CC category, only one GO term (‘plant-type cell wall’) was enriched in the upregulated genes (log2 enrichment value of 0.5). However, among the downregulated genes, terms like ‘exocytic vesicle’, ‘secretory vesicle’, ‘apical plasma membrane’, or ‘pollen tube’ were revealed (log2 enrichment values > 1.6). Finally, within the MF category, GO terms like ‘(+)-neomenthol dehydrogenase activity’, ‘(−)-menthol dehydrogenase activity’, ‘xanthoxin dehydrogenase activity’, ‘chitinase activity’, ‘bis (5′-nucleosyl)-tetraphosphatase (assymetrical) activity’, ‘serine-type endopeptidase inhibitor activity’, ‘bis (5′-adenosyl)-penthaphosphatase activity’, ‘phenylalanine ammonia lyase activity’, or ‘diphosphoric monoester hydrolase activity’ were the sub-functional categories found for the upregulated genes (log2 enrichment values > 2). On the other hand, the downregulated genes showed enriched GO terms related with ‘pectate lyase activity’, ‘Rho GTPase binding’, ‘phosphatidylinositol kinase activity’, ‘Rab GTPase binding’, ‘B-fructofuranosidase activity’, ‘sucrose alpha-glucosidase activity’, ‘solute: proton antiporter activity’, or ‘1-phosphatidylinositol binding’ (log2 enrichment values > 2).

### 2.3. Search of DEGs within QTL Intervals for Autofertility

In the recent high-density genetic map built for the RIL population Vf6 × Vf27 [32], used as well in this study, the authors reported 26 QTLs for traits related with autofertility. The number of markers within the QTL intervals ranged from 1 to 64, and most of them (>80%) matched with a known genome sequence. We checked the differentially expressed genes (DEGs) between AF and AS lines and overlaid these DEGs onto the QTL confidence intervals to find candidate genes associated with these traits. Up to 14 DEGs (transcriptome sequences) matched with at least one marker in the interval of eight different QTLs (Supplementary File S4). The corresponding genome sequences were selected and blasted (BLASTx) to determine the putative function of these genes (Table 2). One of these DEGs specifically matched with the significant marker associated with the QTL corresponding to the PSC2008/09 trait, related to the pod set in chromosome IV. This marker was downregulated in autofertile lines and was identified as AT3G07960, a phosphatidylinositol 4-phosphate 5-kinase (PIP5K6) protein (Table 2).

### 2.4. Quantitative Real-Time PCR Analysis

To corroborate the relative expression levels obtained by RNA-Seq, the transcript levels of seven selected DEGs that were highly up- or downregulated were analyzed by quantitative real-time PCR (qRT-PCR) in the parental lines. Four of them were included in the QTL intervals of some autofertility traits, including those related to the rupture of the stigmatic cuticle. Vf1g128360 was upregulated in the autofertile line (Vf27), whereas Vf4g039440, Vf4g042680, and Vf6g026920 were downregulated in the autofertile line (Supplementary File S5). The expression profile of the DEGs analyzed by qRT-PCR was consistent with the original RNA-Seq data, indicating the reliability of the data obtained in this study.

## 3. Discussion

### 3.1. Sequencing, Assembly, and Annotation

In this study, more than 1000 million clean reads have been obtained from the transcriptome sequencing of the styles and stigmas of faba bean flowers using the Illumina Novaseq platform. A minimum of 42 million clean reads per sample and a maximum of 67 million were obtained (with the exception of the sample Vf27.18 sequenced at a higher depth that produced more than 121 million clean reads). The de novo assembly resulted in 76,269 contigs, with an N50 of 2387 bp and an average contig length of 982.9 bp. Compared to other recent faba bean transcriptome studies [36,37,38,39,40], the number of unigenes acquired in our study was intermediate, but the N50 value was higher. All these previous studies used the Illumina platform to sequence the libraries, and most of them performed the assembly with the Trinity v. 2.8.4 software.

Concerning the annotation, only 34,421 out of 76,269 sequences (45.1%) could be annotated with at least one GO term using the TRAPID v. 2.0 software. This percentage was relatively low despite the several software assayed to analyze the data. The percentage of annotation for the abovementioned faba bean transcriptomes varied depending on the databases used, ranging from 40.8% [39] to 71.5% [38] for unigenes annotated against the NCBI non-redundant (Nr) protein sequence database, which is usually the one to obtain the highest annotation percentages. Poor annotation rates were also reported in the stigma transcriptomes from different species. Thus, Wang et al. [41] annotated 43.7% of the sequences in jasmine, He et al. [42] annotated 53.8% of the sequences in *Camellia oleifera*, and Quiapim et al. [43] reported no hits or known function matches in 52.1% of the *Nicotiana* novel sequences after BLASTx searches. Additionally, in a recent proteome and transcriptome analysis using stigmas and pollen from *Brassica*, Robinson et al. [7] pointed out that many of the proteins revealed in their study still have no known biological roles. This relatively low percentage of annotation or identification may be a result of the scarce genetic information available for flower stigmas and the underlying processes beyond the genetics of incompatibility systems. In addition, many of these sequences may correspond to rapidly evolving species-specific genes involved in sexual reproduction which display high diversity in order to maintain species boundaries [5,8].

Previous works on the pistil composition highlight broad similarities between species with wet and dry stigmas for some functional categories such as ‘defense and stress response’, ‘carbohydrate and energy metabolism’, ‘protein metabolism and folding’, ‘cell wall remodeling’, ‘signal transduction’, ‘photosynthesis’, or ‘lipid metabolism’ [10]. Our analysis also showed numerous GO annotations related to these broad categories like ‘response to stress’ or other types of stimuli, ‘carbohydrate metabolic process’, ‘protein metabolic process’, ‘signal transduction’, or ‘lipid metabolic process’ (Figure 1). Similarly, the KEGGs enrichment analysis also showed pathways related to these categories, like ‘plant–pathogen interaction’, ‘pyruvate metabolism’, ‘biosynthesis of amino acids’, ‘MAPK signaling pathway’, ‘Phosphatidylinositol signaling system’, ‘fatty acid degradation’, etc. (Figure 2).

One of the goals of this study was to identify genes differentially expressed between autofertile and autosterile lines. The KEGGs enrichment analysis revealed several statistically enriched pathways in this set of genes. The upregulated DEGs in the autofertile lines were mostly enriched in pathways related to the synthesis of amino acids, such as ‘Selenocompound metabolism’, ‘Valine, Leucine and Isoleucine biosynthesis’, or ‘Arginine biosynthesis’. Another highly enriched pathway was ‘One carbon pool by folate’. Folates act as donors and acceptors in one-carbon transfer reactions and are involved in the synthesis of important biomolecules such as amino acids, nucleic acids, and vitamin B5 (reviewed in [44]). But, it has also been related to stress responses, and, among them, the response to oxidative stress.

Metabolic pathways related to the synthesis or degradation of terpenoids were also highly enriched in the DEGs. ‘Monoterpenoid biosynthesis’ was enriched in the upregulated genes, whereas ‘Limonene and pinene degradation’ (two monoterpenes) was notably enriched in downregulated genes. In addition, ‘Diterpenoid biosynthesis’ was significantly enriched in both up- and downregulated genes. Many monoterpenoids are volatile compounds and can be found in the essential oils of many plants. The biological functions of many of them are related to the attraction or repellent of insects such as pollinators or herbivores [45]. For example, three volatile monoterpenes (linalool, limonene, and β-pinene) can be identified by wasps from receptive female flowers of figs, which is the only stage receptive to pollinators [46]. *Arabidopsis thaliana* mutant plants that lacked the emission of a volatile sesquiterpene showed greater bacterial growth on their stigmas than the flowers of wild-type plants did [47]. On the other hand, it has been seen that the beetle *Bruchus rufimanus*, an important faba bean pest, responds to floral volatiles in physiological and behavioral experiments, though the beetle did not necessarily pollinate the flowers [48]. Therefore, terpenes play important roles in defense against biotic interactions, as could be the case in faba bean flowers, with the already receptive stigmas emitting volatiles of monoterpenes for both, protecting against pathogens and attracting pollinators. In addition to terpenoid metabolism, the ‘Phenylpropanoid biosynthesis’ was also enriched in the styles and stigmas of faba bean flowers. Phenylpropanoids are also part of the secondary metabolism of plants, contributing to all aspects of plant responses to abiotic and biotic stimuli [49].

Regarding signal transduction, several routes stand up in the enrichment analysis, highlighting the importance of the recognition of different stimuli in stigmas. The ‘MAPK signaling pathway’ was enriched in both up- and downregulated genes, whereas the ‘Phosphatidylinositol signaling system’ and ‘AGE/RAGE signaling pathway’ were notably enriched in the downregulated genes. A Mitogen-activated protein kinases (MAPKs) cascade is required for maintaining the stigma receptivity to accept compatible pollen in *Arabidopsis*. MAPKs converge in the receptivity factor Exo70A1, a member of the exocyst complex. The phosphorylation of Exo70A1 by MAPKs regulates pollen hydration and germination through exocytosis in *Brassica* and *Arabidopsis* species [50]. As reported by McInnis et al. [51], the accumulation of reactive oxygen species (ROS) in mature stigmas in a constitutive way suggests that ROS might be an upstream candidate signal as they are known to activate these kinases. Regarding the AGE-RAGE signaling pathway, it is better known in animals than plants. AGEs is the acronym of advanced glycation end products. AGEs are involved in the pathogenesis of diabetes mellitus, Alzheimer’s disease, aging, and are also involved in the thermal processing of foods. Multiple membrane and soluble proteins have been annotated as receptors for glycation products in mammals (e.g., RAGEs). Upon interaction with receptors, AGEs trigger an inflammatory response by the activation of mitogen-activated protein kinase (MAPK-), janus kinases (JAC-), and mitogen-activated protein kinases/extracellular signal-regulated kinases MAPK/ERK signaling pathways [52]. However, the role of glycation in plants is poorly understood, and two main aspects are proposed: glycation as a marker of aging, senescence, and tag for protein degradation and as a possible mechanism of signaling (reviewed in [53]).

On the other hand, the ‘Phosphatidylinositol signaling pathway’ was enriched in downregulated DEGs. Inositol phospholipid compounds (such as IP3 and DAG) on the cell membrane are important secondary messengers involved in signal transduction [54]. For example, many components of the phosphatidylinositol signaling system participate in vacuolar diversification during pollen development and vesicle transport in pollen tube growth. A good regulation of phosphatidylinositol-4-phosphate and phosphatidylinositol 4,5-bisphosphate pools is necessary for polarized secretion in plants (reviewed in [55]). Gradients of these compounds have been observed in root hairs and pollen tubes where they are linked to polarized secretion [56,57]. Since the stigmatic papillae in faba bean are specialized in secreting the exudates, functions related with vesicle transport are expected to be found in this tissue.

### 3.2. DEGs within QTL Intervals Previously Described for Autofertility

From the significant markers found to be associated with autofertility traits by QTL analysis [32], one DEG (Vf4g039440) matched, in chromosome IV, with the significant QTL marker for PSC_2008/09, related to pod set under insect proof cages. This transcript was identified as a phosphatidylinositol 4-phosphate 5-kinase 6-like (PIP5K6) protein (Table 2) and was downregulated in autofertile lines (corroborated also by RT-qPCR). PIP5Ks are required for phosphatidylinositol 4,5-bisphosphate [PI(4,5)P_2_] formation, which interacts with a wide variety of proteins modulating their molecular functions (reviewed in [58]). For example, it has been recently demonstrated that PI(4,5)P_2_ production by PIP5K4, PIP5K5, and PIP5K6 is essential for pollen germination by the establishment of the germination polarity in a pollen grain [59]. The role of phosphoinositides in membrane trafficking has been demonstrated in growing pollen tubes. The suppression of *PIP5K6* expression impaired clathrin-dependent endocytosis and slow tube elongation; however, *PIP5K6* overexpression showed plasma membrane invagination and the formation of tip branches due to a higher rate of endocytosis [57]. Beyond the role of PI(4,5)P_2_ in pollen germination or pollen tube elongation, this phosphoinositide and its production has also been studied in response to pathogens. The PI(4,5)P_2_ levels were mildly reduced after the flg22 treatment of *Arabidopsis* plants, which was also related with a reduction in the endocytosis of different plant defense proteins such as the NADPH-oxidase RbohD. Reduced RbohD-endocytosis was correlated with an increase in ROS production [60].

Of particular interest in this study were the genes involved in the receptivity of the stigmas and autofertility. Successful pollination, fertilization, and seed set depend upon the receptivity of stigmas during the few days following anthesis. Some DEGs identified in the transcriptome matched within the genetic intervals of QTLs related with the rupture of the stigmatic cuticle [32], such as RUPTL (rupture length of stigma cuticle) in chr. I and %RUPTAREA (percentage of ruptured area) in chr. VI (see [31] for further details about these measures). In the genetic interval of the RUPTL QTL, we found one DEG (Vf1g128360) upregulated in autofertile lines and identified as a Proline dehydrogenase 2 (ProDH2) protein, which is involved in proline catabolism. Beyond its role in protein biosynthesis, regulated proline accumulation occurs in plant tissues in response to developmental and environmental stimuli. There are two *ProDH*s genes (*ProDH1* and *ProDH2*) in *A. thaliana* which encode for homologous and functional isoenzymes; however, they show distinctive expression patterns. *ProDH1* shows greater expression in pollen and stigmas and is expressed in most developmental stages and tissues; however, *ProDH2* shows low expression levels and is mostly expressed at vascular tissues and senescent leaves [61]. Proline degradation occurs in the mitochondria, where it is converted to glutamate, and notably ROS are generated as by-products of mitochondrial respiration [62]. Recent studies have reported a regulatory role in the interaction between proline metabolism and ROS production in different tissues and processes [63,64]. Therefore, here we find a new possible relation between proline metabolism and the rupture of the stigmatic cuticle, which is related with the receptivity and the presence of ROS in the stigmas of *Vicia faba*.

The second QTL related to the rupture of the stigmatic cuticle (%RUPTAREA), located in chr. VI, also included a DEG within its genetic interval. This DEG (Vf6g026920) was identified as an ATP-binding cassette (ABC) transporter G family member 28. ABC transporters in plants are more numerous than in other organisms and are classified into eight subfamilies: A–G and I. They are composed of nucleotide-binding domains (highly conserved) and transmembrane domains, with the latter being very variable, allowing for the transport of different substrates. Full-size ABC proteins can work like transporters themselves, but half-size transporters can form complexes to perform their functions. Many full-size ABCG transporters are implicated in defense against biotic stresses (e.g., see [65]). Two half-size ABCG transporters of *M. truncatula* that are present in peri-arbuscular membranes are implicated in arbuscule development in mycorrhizal symbiosis [66]. Another two half-size ABCG transporters are implicated in the stigma exertion in *Medicago* [67]. AtABCG28 is a critical half-size transporter of *A. thaliana* that establishes the correct level of reactive oxygen species (ROS) at the pollen tube and root tips. AtABCG28 is specifically localized to the membranes of secretory vesicles and expressed in mature pollen and growing pollen tubes. It is involved in sequestering polyamines (source of ROS) into the vesicles that move and fuse to the growing tip [68]. Since these QTLs are implicated in the rupture of the stigmatic cuticle, which is also related with the presence of exudate and receptivity of the stigma, high levels of ROS are expected in this tissue. Therefore, the regulation of ROS levels and transport of these substances are important to maintain the correct cellular functions and prevent cell damage.

## 4. Materials and Methods

### 4.1. Plant Materials and Sample Collection

The recombinant inbred line (RIL) faba bean population of 124 individuals derived from the cross between lines Vf6 and Vf27 has been previously used for the localization of QTLs related to autofertility, dehiscence, flowering time, and other yield-related traits [31,69,70]. The parental line Vf6 is a highly autosterile and asynaptic line, whereas Vf27 is considered highly autofertile. The materials selected in this study were six genotypes from this RIL population: the two parental lines (Vf6 and Vf27), two highly autosterile RILs (RIL19 and RIL96), and two highly autofertile RILs (RIL14 and RIL44).

Plants were grown in 5 L pots under controlled conditions (22 °C, 14 h day–10 h dark). At the peak of flowering production for each line, flowers previous to anthesis were collected over several days and dissected to extract the flower style. Style samples were immediately frozen in liquid N_2_ and stored at −80 °C until the RNA extraction was performed.

### 4.2. RNA Extraction, Sequencing, and De Novo Assembly

The total RNA of approximately 100 styles per sample was extracted using TRIZOL reagent (St. Louis, MO, USA) with the Direct-zolTM RNA MiniPrep Kit (Zymo Research Corp, Tustin, CA, USA) according to the manufacturer’s instructions. A total of 18 samples were finally prepared consisting of three replicates for each of the six genotypes.

Samples were sent to STABVIDA (Caparica, Portugal) for quality control, library construction (with a Stranded mRNA Library Preparation Kit), sequencing (Illumina Novaseq, 150 bp paired-end reads), and assembly. Raw sequences were trimmed to generate high-quality reads. For each original read, the following parameters were applied: a quality trimming based on a quality score of 0.01 (error probability), a limit of the length of ambiguity of 2 nt, and a minimum read length of 30 nt. Sample Vf27.18 was sequenced at a higher depth, and the high-quality sequence reads were used for the de novo assembly in Trinity 2.8.4 [71]. The assembled transcriptome of sample Vf27.18 was used as the reference sequence for the expression analysis. The raw reads of this study have been deposited into the NCBI Sequence Read Archive (SRA) database under the accession number PRJNA1044928.

### 4.3. Annotation and Differential Expression Analysis

Contigs obtained from the assembly of sample Vf27.18 were annotated with TRAPID [72], a web application for taxonomic and functional analysis, using PLAZA 4.5 dicots [73] as the database and clade Papilionoideae as a similarity search database with a threshold of 10-5. GO graphs were summarized according to the GO slims categories for plants.

The high-quality reads from each sample were mapped against the de novo assembled transcriptome reference. A minimum similarity and length fraction of 0.8 were used as parameters to consider a correctly mapped read. The differential expression analysis was performed with edgeR package [74] in R v. 4.2.1 [75]. The identified differentially expressed genes (DEGs) were filtered using a fold change value of >2 or <−2 and an FDR (False Discovery Rate) *p*-value < 0.05 as thresholds.

In order to identify significant metabolic pathways correlated with putative autofertility genes, we focused on the subset of DEGs existing between all the autosterile and all the autofertile samples. A KEGGs pathway enrichment analysis of the DEGs was performed using the KEGGs pathway database in KOBAS-I and the more recent KEGGs Orthology-Based Annotation System [76] using *Medicago truncatula* as the reference database. A *p*-value < 0.05 was considered to indicate significant over-representation of a certain KEGG pathway. We also performed a gene ontology (GO) enrichment analysis in TRAPID, which determines the over-representation of a certain GO term compared to the background frequency (i.e., Papilionoideae dataset). The Benjamini and Hochberg correction was further applied to control multiple testing and decrease the FDR (q-value < 0.05 was established as a threshold to determine if the GO term was enriched in the dataset).

### 4.4. Search of DEGs within QTL Intervals Previously Described for Autofertility

To identify candidate genes controlling autofertility, we combined the results of previous QTL mapping [32] with the transcriptome data. Molecular markers falling within the QTL intervals were selected, and the corresponding nucleotide sequences were extracted from ‘Vfaba_v2’ 60k SNP Array [77,78]. Marker sequences were first aligned against the faba bean genome [79], and the genome sequences were then aligned against the transcriptome sequences of the DEGs. Those DEGs falling within the QTL intervals were identified by BLASTx.

### 4.5. Quantitative Real-Time PCR Analysis

A quantitative real-time PCR (qRT-PCR) analysis was used to corroborate the RNA-Seq results. This experiment was performed with the same RNA extraction of the parental lines (Vf6 and Vf27) used for the RNA-Seq experiment. For cDNA synthesis, 2 µg of total RNA was reverse-transcribed using the iScriptTM cDNA Synthesis Kit (BioRad, Hercules, CA, USA) and diluted to a concentration of 10 ng/µL. The experimental design consisted of a total of 12 samples (2 genotype × 2 technical repetitions × 3 biological repetitions). A pooled sample comprising all the samples considered in the experiment was included for each gene as an inter-run calibrator to detect and correct the inter-run variation. No template controls were included. 

Specific primer pairs for seven DEGs were designed (Supplementary File S6). The DEGs selected were highly up- or downregulated in autofertile vs. autosterile lines, and four of them were overlaid on the QTL intervals related with autofertility traits. Three of them were upregulated (ProDH2 [Vf1g128360], a transmembrane protein [Vf4g117320], and a cytochrome P450 protein [Vf5g094440]) and four were downregulated (B-galactosidase protein [Vf2g121200], a PIP5K6 [Vf4g039440], BUPS1 [Vf4g042680], and a ABCG28 [Vf6g026920]) (Table 2). CYP2 and ELF1A, previously reported as the most stable genes for gene expression normalization in the faba bean experiments [80], were used as the reference genes. 

The qPCR was carried out using the iTaqTM Universal SYBR^®^ Green Supermix on an ABI PRISM 7500 Real-Time PCR System (Applied Biosystems, Foster City, CA, USA). A master mix with a total volume of 11 µL for each PCR run was prepared, containing 4 µL of diluted cDNA (10 ngr/µL), 5 µL of iTaqTM Universal SYBR^®^ Green Supermix (Bio-Rad, Hercules, CA, USA), and a primer pair with a concentration of 0.45 µM each. The thermocycler was programmed to run for ten min at 95 °C, followed by 40 cycles of 15 s at 95 °C and 1 min at 60 °C. Specific amplifications were confirmed by the unique and sharp peak melting curves of the PCR products.

PCR efficiency was determined for all samples by means of the amplicon groups by LinRegPCR program v.1139 using raw normalized (Rn) fluorescence as the input data. Fluorescence was analyzed using 7500 Software v2.0.1 using a threshold value of 0.2 to obtain the Cq (quantification cycle) values for each gene–cDNA combination. The relative gene expression (RGE) was calculated using the advanced quantification model described by Hellemans [81] (Equation (1)), where RQ = E∆Ct, with E = PCR being the efficiency for each primer used to amplify each target gene (TG) and Ct being the number of cycles needed to reach 0.2 arbitrary units of fluorescence. The two reference genes (RG) used for data normalization were CYP2 and ELF1A.

RGE = RQTG/Geomean[RQRG](1)

The RGE values were log-transformed, and ANOVA tests were used to compare the RGE values and obtain significance values with R v. 4.2.1 [75].

## 5. Conclusions

In this study, we used RNA sequencing to check for the first time the differential expression of gene transcripts between faba bean lines differing in autofertility. DEGs were overlaid onto QTLs detected in a recent high-density genetic map to find candidate genes associated with autofertility. The initial challenge in the current study was due to the lack of annotated stigma datasets. Although the experimental validation of the candidate genes has not been performed, DEGs up- and downregulated were identified, and some of them were hypothesized to be related with the traits under study. One DEG matched with the significant marker associated with one QTL related to the pod set and others DEGs mapped in the intervals of QTLs related to the rupture of the stigmatic cuticle. RNAseq combined with QTL mapping is a powerful approach for identifying candidate genes, and the results derived from this work provide an important transcriptomic reference for style-stigma processes to aid our understanding of the molecular mechanisms involved in faba bean fertilization. The new available transcriptomic data and the RIL population used will facilitate the fine mapping of the responsible genes and will provide targets for future study and improvements in the autofertility traits of this crop.

## Figures and Tables

**Table 1:** Summary of the 18 libraries in terms of number of raw reads, number of bases, and number of clean reads obtained. Abbreviations: AF: autofertile; AS: autosterile.

Sample	Raw Reads	Number of Bases (Gb)	Clean Reads
AF27.18	133,319,602	19,997	121,359,412
AF27.28	65,839,438	9875	60,315,740
AF27.5	61,476,838	9221	56,683,402
AF44.1	55,001,874	8250	49,763,678
AF44.4	65,871,434	9880	59,782,290
AF44.14	64,747,566	9712	59,231,922
AF14.2	56,963,200	8544	43,002,584
AF14.5	70,837,140	10,625	65,081,586
AF14.16	63,834,224	9575	58,881,026
AS6.3	73,167,636	10,975	67,520,844
AS6.5	57,074,180	8561	52,288,334
AS6.14	47,358,170	7103	42,875,694
AS96.1	68,186,700	10,228	62,004,170
AS96.9	63,583,396	9537	58,218,214
AS96.12	57,687,624	8653	51,643,386
AS19.2	57,687,624	9500	58,475,436
AS19.3	63,147,560	9472	57,224,622
AS19.9	57,643,612	8646	52,847,570
Total	1,189,079,630		1,077,199,910

**Table 2:** BLASTx searches for the DEGs associated with markers within the QTL intervals for autofertility traits. PSF: pod set field measure; RUPTL: length of stigmatic rupture; PSC: pod set under insect proof cages; PAPL: papilla length; NPAP/STIGL: number of papillae-divided stigma length; %RUPTAREA: percentage of stigmatic ruptured area; SSC: seed set under insect proof cages; OL: ovary length.

Transcript ID	Regulation	Traits	Chr	QTL Marker	Reference Genome Vicia Faba	Organism	Protein ID	Protein Description	Arabidopsis ID
TRINITY_DN9113_c0_g1	UR	PSF_2009/10	CHR1	AX_416823680	1g073840	*Pisum sativum*	XP_050883094.1	protochlorophyllide reductase, chloroplastic	AT5G54190
TRINITY_DN9113_c0_g1	UR	PSF_2009/10	CHR1	AX_181440809	1g073840	*Pisum sativum*	XP_050883094.1	protochlorophyllide reductase, chloroplastic	AT5G54190
TRINITY_DN239_c0_g2	UR	RUPTL	CHR1	AX_181440664	1g128360	*Pisum sativum*	XP_050907114.1	proline dehydrogenase 2, mitochondrial-like	AT5G38710
TRINITY_DN1719_c0_g2	UR	PSC_2008/09	CHR4	AX_416818743	4g037560	*Pisum sativum*	XP_050882017.1	DNA damage-repair/toleration protein DRT100-like	AT3G12610
TRINITY_DN27765_c0_g4	DR	PSC_2008/09	CHR4	AX_416752208	4g039440	*Pisum sativum*	XP_050873114.1	phosphatidylinositol 4-phosphate 5-kinase 6-like	AT3G07960
TRINITY_DN10484_c0_g3	DR	PSC_2008/09	CHR4	AX_416793113	4g042680	*Pisum sativum*	XP_050873201.1	probable receptor-like protein kinase At4g39110	AT4G39110
TRINITY_DN16420_c1_g1	DR	PSC_2008/09	CHR4	AX_181170326	4g042600	*Pisum sativum*	XP_050873193.1	protein CHROMATIN REMODELING 35	AT2G16390
TRINITY_DN21326_c1_g1	DR	PAPL	CHR4	AX_416773786	4g230040	*Pisum sativum*	XP_050920775.1	N6-mAMP deaminase-like	AT4G04880
TRINITY_DN1073_c0_g1	UR	NPAP/STIGL	CHR6	AX_416766831	6g033880	*Trifolium repens*	KAK2436160.1	DNA-binding protein	AT5G59830
TRINITY_DN42106_c0_g1	DR	%RUPTAREA	CHR6	AX_416739703	6g026920	*Pisum sativum*	XP_050899031.1	ABC transporter G family member 28	AT5G60740
TRINITY_DN47868_c0_g1	DR	%RUPTAREA	CHR6	AX_416739703	6g026920	*Pisum sativum*	XP_050899031.1	ABC transporter G family member 28	AT5G60740
TRINITY_DN52070_c0_g1	DR	%RUPTAREA	CHR6	AX_416739703	6g026920	*Pisum sativum*	XP_050899031.1	ABC transporter G family member 28	AT5G60740
TRINITY_DN61450_c0_g1	UR	SSC_2008/09 + OL	CHR6	AX_181176378	6g125280	*Trifolium repens*	KAK2434246.1	plastid movement impaired protein	AT2G01340
TRINITY_DN1763_c0_g2	DR	SSC_2008/09 + OL	CHR6	AX_181445379	6g128440	*Pisum sativum*	XP_050893915.1	xyloglucan endotransglucosylase/hydrolase 2	AT3G23730
TRINITY_DN1344_c0_g4	DR	SSC_2008/09 + OL	CHR6	AX_181445379	6g128440	*Pisum sativum*	XP_050893915.1	xyloglucan endotransglucosylase/hydrolase 2	AT3G23730

## Data Availability

Raw reads of this study have been deposited into the NCBI Sequence Read Archive (SRA) database under the accession number PRJNA1044928 (https://www.ncbi.nlm.nih.gov/sra/PRJNA1044928).

## Supplementary Materials

The following supporting information can be downloaded at: https://www.mdpi.com/article/10.3390/plants13111443/s1, File S1: Heat map of the differentially expressed genes between autofertile (right) and autosterile lines (left). Low expression levels are depicted in blue whereas high expression levels are depicted in red.; File S2: Gene ontology (GO) functional classification of the differentially expressed genes (DEGs) between autofertile and autosterile lines. Histogram of the main transcripts annotated to specific GO categories: Biological Processes, Cellular Components and Molecular Function. The x-axis represents the GO term and the y-axis represents the number of genes annotated.; File S3: GO enrichment analysis performed in TRAPID of the differentially expressed genes of autofertile vs. autosterile lines. Highlighted in green GO terms related with upregulated genes and highlighted in red GO terms related with downregulated genes.; File S4: QTLs for autofertility traits in the RIL population Vf6 x Vf27 (Aguilar-Benitez et al. manuscript in preparation), number of markers within each of the QTLs intervals, markers associated with a known genome sequence and number of markers associated with a transcriptome DEGs. PSF: pod set field measure; PSC: pod set under insect proof cages; PST: pod set after tripping treatment; SSF: seed set field measure; %RUPT: percentage of rupture of stigma surface; RUPTL: length of the stigmatic rupture; OL/FL: ovary length divided by flower length; SL/FL: style length divided by flower length; STIGL: stigma length; SL: style length; PAPL: papilla length; %RUPTAREA: percentage of ruptured stigmatic area; NPAP/STIGL: number of papillae divided stigma length; SSC: seed set under insect proof cages; OL: ovary length; TOTALS: mean size of pollen grains; NORMAL%: percentage of pollen normal; RATIO_PSIZE: ratio of pollen size (NORMALS/TOTALS).; File S5: Expression values for the parental lines Vf27 (autofertile) and Vf6 (autosterile) of seven genes detected by RNA-Seq (bar graph, left y-axis) and qRT-PCR (dot and lines graph, right y-axis). RNA-Seq values have been log2 transformed. Statistically significant differences were found for all the genes.; File S6: Information on qRT-PCR primers used for verification of RNA-Seq data. PCR efficiency and PCR product Tm data represent mean values ± sd.
