# Supplementary material for: Transcriptome Analysis of Stigmas of Vicia faba L. Flowers
Source: Plants (Basel). 2024 May 23;13(11):1443. doi: 10.3390/plants13111443 (PMC11175038; doi:10.3390/plants13111443)

Supplementary file S1. Heat map of the differentially expressed genes between autofertile (right) and autosterile lines (left). Low expression levels are depicted in blue whereas high expression levels are depicted in red.

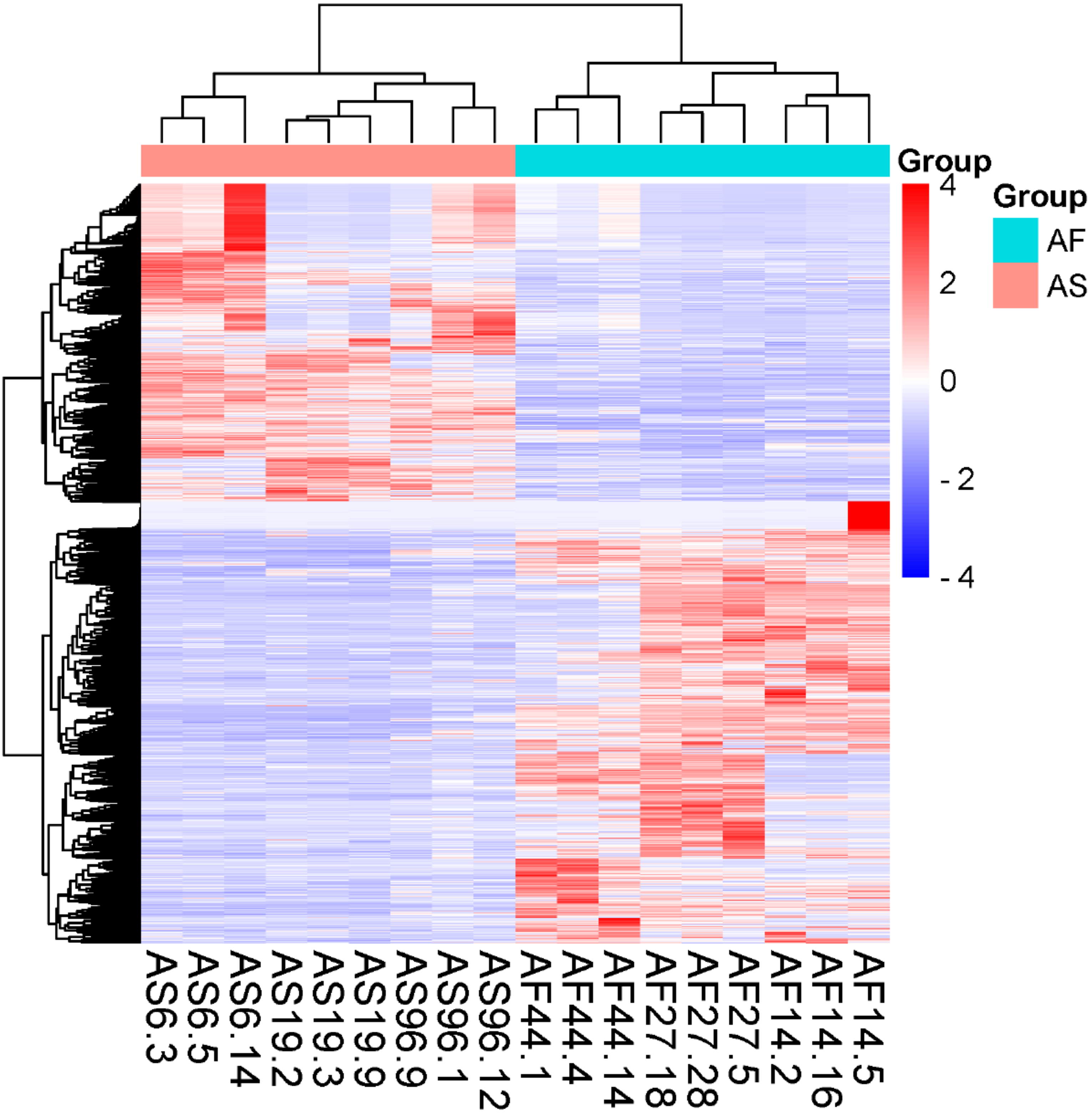

Supplement: Supplementary file 1 [file plants-13-01443-s001.zip › Supplementary_file_S1_Heat_map_DEGs.pdf]
