# Supplementary material for: Transcriptome Analysis of Stigmas of Vicia faba L. Flowers
Source: Plants (Basel). 2024 May 23;13(11):1443. doi: 10.3390/plants13111443 (PMC11175038; doi:10.3390/plants13111443)

Supplementary file S2. Gene ontology (GO) functional classification of the differentially expressed genes (DEGs) between autofertile and autosterile lines. Histogram of the main transcripts annotated to specific GO categories: Biological Processes, Cellular Components and Molecular Function. The x-axis represents the GO term and the y-axis represents the number of genes annotated.

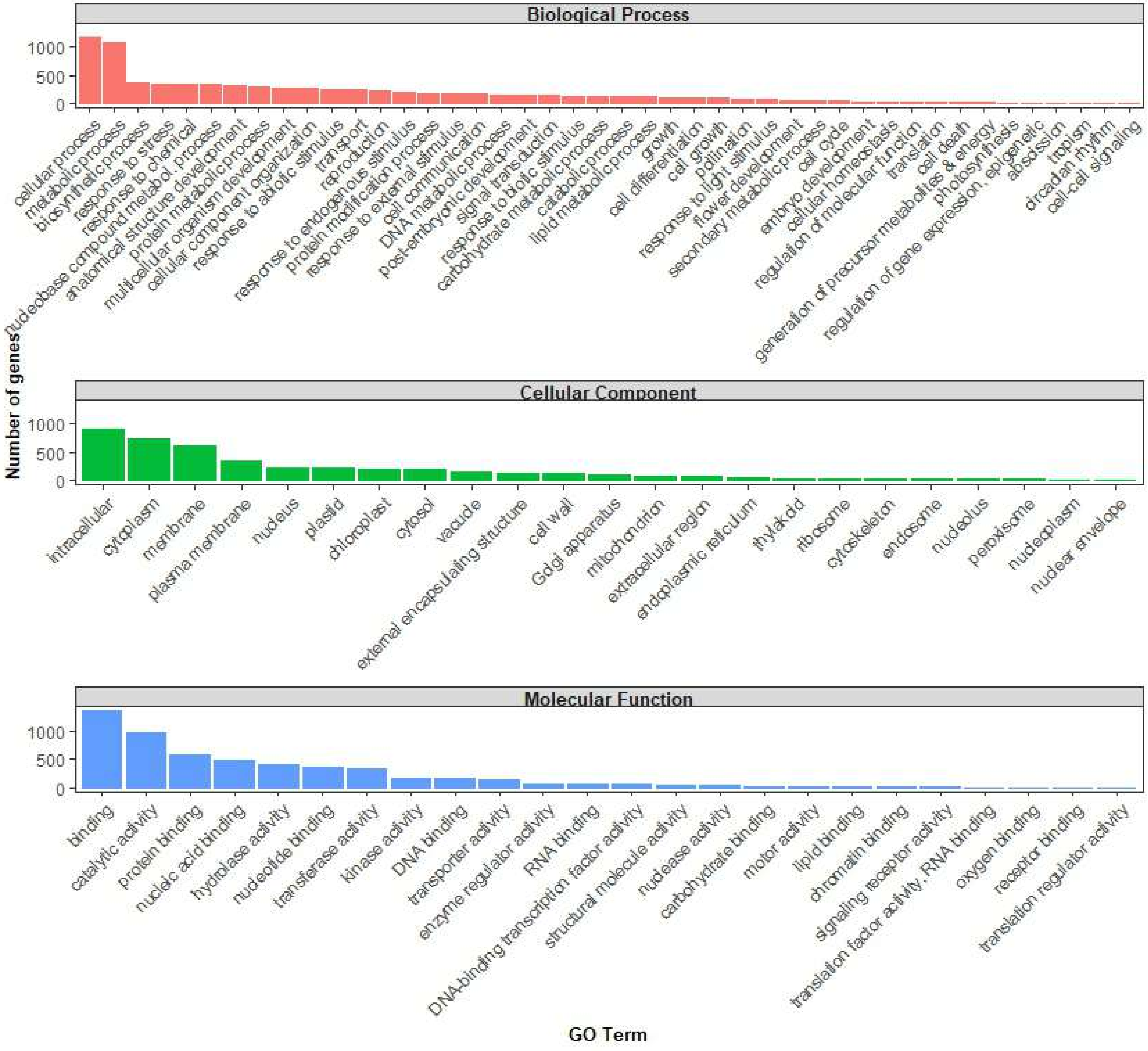

Supplement: Supplementary file 1 [file plants-13-01443-s001.zip › Supplementary_file_S2.pdf]
