# Supplementary material for: Transcriptome Analysis of Stigmas of Vicia faba L. Flowers
Source: Plants (Basel). 2024 May 23;13(11):1443. doi: 10.3390/plants13111443 (PMC11175038; doi:10.3390/plants13111443)

Supplementary file S5. Expression values for the parental lines Vf27 (autofertile) and Vf6 (autosterile) of seven genes detected by RNA-Seq (bar graph, left y-axis) and qRT-PCR (dot and lines graph, right y-axis). RNA-Seq values have been log2 transformed. Statistically significant differences were found for all the genes.

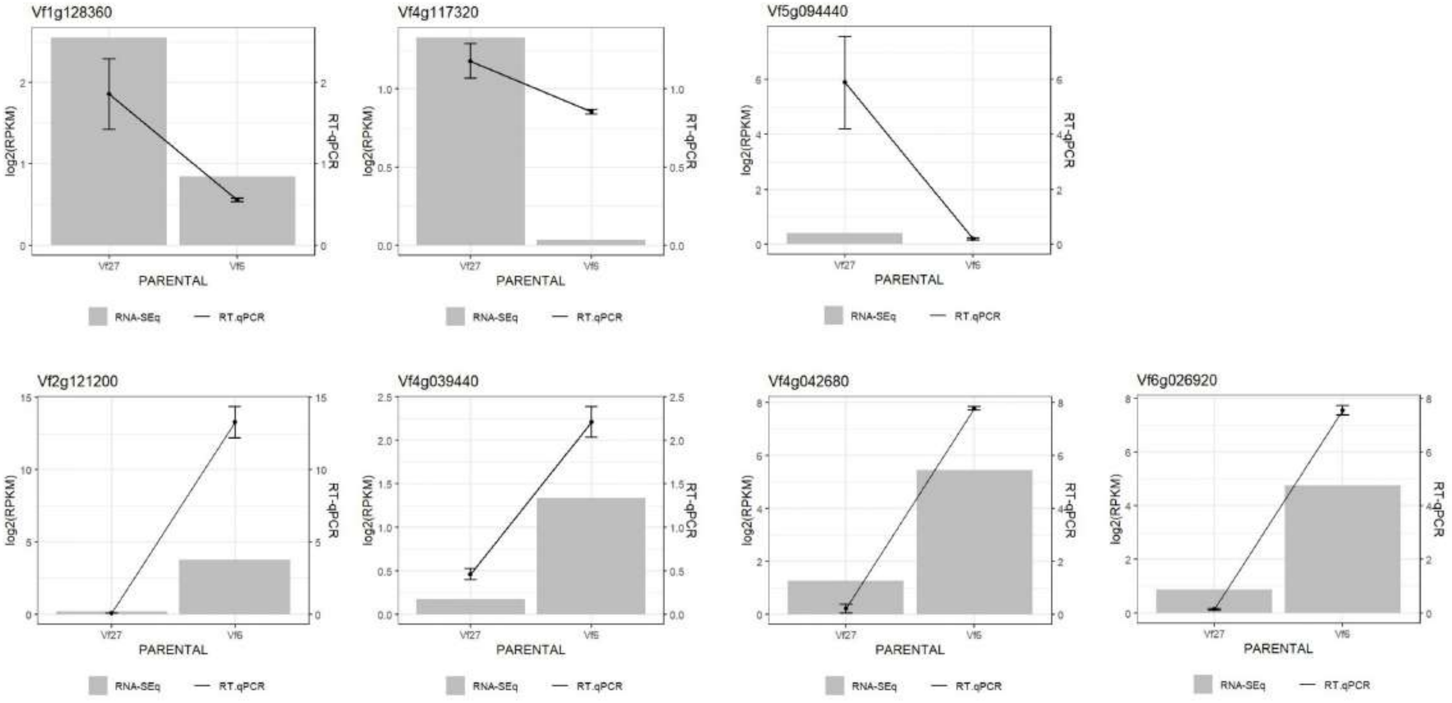

Supplement: Supplementary file 1 [file plants-13-01443-s001.zip › Supplementary_file_S5.pdf]
